# Effects of *Tenebrio molitor* and *Zophobas Morio* larvae meal supplementation on growth performance, carcass traits, and gut histomorphology in Japanese quails

**DOI:** 10.1016/j.psj.2025.105803

**Published:** 2025-09-05

**Authors:** Hanan Al-Khalaifah, Zeeshan Ahmad, Rafi Ullah, Ziaul Islam, Asad Sultan, Ziaul Islam, Ala Abudabos, Shabana Naz, Rifat Ullah Khan, Ibrahim A. Alhidary

**Affiliations:** a Environment and Life Sciences Research Center, Kuwait Institute for Scientific Research, Kuwait 10 City, Kuwait; b Department of Poultry Science, Faculty of Animal Husbandry and Veterinary Sciences, The University of Agriculture, Peshawar, Pakistan; c Department of Basic and Applied Zoology, Shaheed Benazir Bhutto University, Sheringal, Upper Dir, Pakistan; d Institute of zoological sciences, University of Peshawar-Pakistan, Peshawar, Pakistan; e Department of Food and Animal Sciences, College of Agriculture, Tennessee State University, Nashville, TN 37209, United States; f Department of Zoology, Government College University, Faisalabad, Pakistan; g Physiology Lab, College of Veterinary Sciences, Faculty of Animal Husbandry and Veterinary Sciences, The University of Agriculture, Peshawar, Pakistan; h Department of Animal Production, College of Food and Agriculture Science, King Saud University, Riyadh, Saudi Arabia

**Keywords:** Japanese quail, Insect meal supplementation, Growth performance, Gut histomorphology

## Abstract

This study investigated the effects of mealworm (*Tenebrio molitor*) and superworm (Zophobas Morio) meal supplementation on the growth performance, carcass traits, and gut histomorphology of Japanese quails. A total of 240 day-old quail chicks were randomly assigned to four dietary treatments in a Completely Randomized Design (CRD) with five replicates per treatment. The treatments included: a control diet without insect meal (A), a diet with 3 g/kg mealworm meal (B), a diet with 3 g/kg superworm meal (C), and a diet combining 3 g/kg mealworm and 3 g/kg superworm meal (D). Feed and water were provided ad libitum for 35 days. Growth performance parameters, carcass traits, and gut morphology were evaluated. Results indicated that insect meal supplementation significantly influenced feed intake, body weight gain, and feed conversion efficiency. The combination (MW+SW) diet reduced feed intake while enhancing growth and feed efficiency. Carcass traits analysis revealed that the MW+SW diet significantly increased dressing percentage. Gut histomorphology assessment showed improved villus height and crypt depth, suggesting enhanced nutrient absorption. Overall, the combination diet (MW+SW) demonstrated the most beneficial effects, highlighting its potential as an alternative protein source in quail nutrition.

## Introduction

Recent advancements in poultry nutrition have highlighted the potential of alternative feed additives to improve growth and health (Magnoli et al., 2024; Nouri et al., 2024; Hafeez et al., 2025; Khan et al., 2025). Fermentation and enzyme treatments have been shown to enhance nutrient content and digestibility (Devi et al., 2023; Mirnawati et al., 2023; Boonmee et al., 2024; Dinasarki et al., 2024; Othman et al., 2024). These approaches also support immune response, stress tolerance, and gut microbial balance (Al-Suwailem et al., 2024; Habib et al., 2024; Sarsembayeva et al., 2025; Sultana et al., 2025). Building on this foundation, insect meals are now being explored as sustainable alternatives with comparable or superior benefits in poultry diets (Flis et al., 2024).

In recent years, edible insects have gained significant attention as an environmentally friendly and nutritionally rich protein source for poultry production (Ajmal et al., 2023). Among the different insect species, superworms (Zophobas Morio) and mealworms (*Tenebrio molitor*) have been extensively studied due to their high protein, fat, and micronutrient contents (Van Huis, 2021). These insects are rich in essential amino acids, polyunsaturated fatty acids, vitamins, and minerals, making them a viable substitute for conventional protein sources such as soybean meal and fish meal in poultry diets (Dragojlović et al., 2022). As global concerns over environmental sustainability and feed resource scarcity continue to rise, incorporating insect meals into poultry diets offers a promising solution to reduce dependency on traditional feedstuffs while ensuring optimal bird performance and health (Elahi et al., 2022; Shaukat et al., 2023).

Superworms and mealworms differ in their nutrient composition, which may influence poultry growth, feed efficiency, and overall health. Superworms have a higher fat and fiber content compared to mealworms, which contain a more balanced protein-to-fat ratio (Pietras et al., 2021). Studies suggest that the inclusion of insect meal in poultry diets improves feed conversion ratios (FCR), enhances nutrient digestibility, and supports gut health (Biasato et al., 2018). Moreover, these insects possess bioactive compounds, such as antimicrobial peptides, which may offer additional health benefits by enhancing immune responses and reducing pathogen load in poultry intestines (Elahi et al., 2022). The sustainability of poultry production heavily depends on feed efficiency and ingredient availability (Rasool et al., 2023). Conventional protein sources, such as soybean meal, contribute to deforestation, land degradation, and increased carbon emissions (Van Huis, 2021). Conversely, insect farming requires less land, water, and feed resources while producing significantly lower greenhouse gas emissions (Dragojlović et al., 2022). Incorporating insect meals into poultry diets could mitigate environmental concerns and contribute to a circular bio economy by utilizing organic waste to rear insects. Moreover, economic viability is a crucial factor in determining the feasibility of using insect meal as poultry feed ingredient. Apart from nutritional benefits, insect-based diets have been linked to improved poultry health and welfare. The chitin present in insect exoskeletons acts as a prebiotic, promoting beneficial gut microbiota and enhancing immune function (Elahi et al., 2022). Furthermore, insect-derived lipids contain high levels of lauric acid, which exhibits antimicrobial properties and may reduce the prevalence of pathogenic bacteria such as *Salmonella* and *Escherichia coli* in poultry intestines (Biasato et al., 2018). Although previous studies have examined the use of insect meals in poultry diets, there is limited comparative data on superworm and mealworm supplementation, specifically in Japanese quail. The present study aims to fill this knowledge gap by investigating their impact on growth performance, carcass traits, and intestinal histomorphology.

## Materials and methods

### Birds and experimental design

A total of 240 day-old Japanese quail (Coturnix japonica) chicks were procured from a local market and reared under standardized management conditions. The birds were distributed in a Completely Randomized Design (CRD) into four dietary treatment groups, each consisting of 60 quail chicks subdivided into five replicates (*n* = 12 per replicate). The trial spanned 35 days, during which feed and water were provided ad libitum. Housing consisted of floor pens lined with wood shavings as bedding, and the birds were maintained under continuous 24-hour lighting. Strict biosecurity protocols were implemented to mitigate disease risks, including facility disinfection, restricted farm access, and adherence to hygiene standards, thereby ensuring optimal flock health and production performance.

### Dietary treatments

Four dietary treatments were evaluated: Control (basal diet); 3 g/kg Tenebrio molitor meal (TM); 3 g/kg Zophobas morio meal (ZM); and combined TM+ZM (3 g/kg each, 6 g/kg total). Diets were formulated (Table 1), with insect meals (proximate composition in Table 2) defatted, ground (<500 μm), and uniformly incorporated. All diets maintained consistent protein (24 %) and energy (2900 kcal/kg) levels.

**Table 1:** Feed formulation and chemical analysis.

Ingredients (%)	Control diet	Mealworm added diet	Superworm added diet	Mealworm + Superworm
Corn	55.00	55.00	55.0	55.0
Soybean meal (44 %)	30.00	29.0	28.0	28.0
Fish meal	5.00	5.00	5.0	5.0
Mealworm	-	0.3 (3g/kg)	-	0.3 (3g/kg)
Superworm	-	-	0.3 (3g/kg)	0.3 (3g/kg)
Vegetable oil	3.00	2.50	2.5	2.4
Dicaclium phosphate	1.5	1.5	1.5	1.5
Limestone	1.2	1.2	1.2	1.2
DL-Methionine	0.2	0.1	0.1	0.8
L-Lysine	0.1	0.05	0.05	0.05
Salt	0.3	0.3	0.3	0.3
Vitamins-minerals premix	0.5	0.5	0.5	0.5
Biochemical analysis				
Crude protein, %	22.0	22.0	22.0	22.2
Metabolizable energy, kcal/kg	2900	2890.0	2910.0	2910.0
Crude Fiber (%)	4.0	4.2	4.1	4.2
Ether Extract (%)	5.5	5.6	5.4	5.5
Calcium (%)	1.00	1.00	1.00	1.00
Phosphorus (%)	0.45	0.45	0.45	0.45
Methionine (%)	0.5	0.5	0.5	0.55
Lysine (%)	1.10	1.10	1.00	1.05
Threonine (%)	0.8	0.8	0.7	0.9
Arginine (%)	1.3	1.3	1.2	1.25

^1^Provided per kg of diet: vitamain A, 8000 IU; vitamain D3,2000 IU; vitamain E,15 IU; vitamain K3, 1.5 mg; thiamine, 2 mg; riboflavin, 5 mg; pyridoxine, 5 mg; vitamain B12, 0.02 mg; folic acid, 0.7 mg; nicotinic acid, 40 mg; pantothenic acid, 12 mg; biotin, 0.2 mg b Provided per kg of diet: copper, 10 mg (as copper sulfate); iron, 90 mg (as ferrous sulfate); manganese, 100 mg (as manganese sulfate); zinc, 100 mg (as zinc sulfate); selenium, 0.3 mg (as sodium selenite); iodine, 0.5 mg (as calcium iodate).

**Table 2:** Proximate analysis of meal worm and super worm.

Parameters	Meal worm (%)	Super worm (%)
Moisture	67.7	66.4
Crude Protein	49.4	51.5
Crude Fat	31.5	33.8
Crude Fiber	5.4	6.3
Ash	6.6	6.5
Nitrogen-Free Extract	15.8	16.1

### Proximate methodology for mealworm **(Tenebrio molitor)** and superworm (**Zophobas morio**)

The proximate composition of Tenebrio molitor and Zophobas morio larvae was analyzed following standard AOAC (2000) methods to determine moisture (oven drying at 105°C), crude protein (Kjeldahl method, *N* × 6.25), crude fat (Soxhlet extraction with petroleum ether), crude fiber (acid-base digestion), ash (muffle furnace incineration at 550°C), and nitrogen-free extract (NFE; calculated by difference). Prior to analysis, larvae were oven-dried and mechanically ground, and lipid content was reduced using Soxhlet extraction with petroleum ether, yielding partially defatted insect meals. This defatting step was performed to minimize excess fat and provide more consistent nutrient profiles for dietary inclusion.

### Growth performance

Growth performance parameters were monitored throughout the trial period. Individual body weight (BW) was measured weekly using a calibrated digital balance (±0.1 g precision), while pen-based feed intake (FI) was determined gravimetrically by subtracting residual feed weight from feed provided. Body weight gain (BWG) was calculated as: BWG (g/bird) = Final BW - Initial BW. Feed conversion ratio (FCR) was computed as: FCR = Total FI (g)/Total BWG (g), with adjustments made for mortality by including the weight of deceased birds in calculations. Mortality was recorded daily during morning time, and all dead birds were weighed to correct FI data (Sultan et al., 2024).

### Carcass traits analysis

At the end of the 35-day experimental period, two birds per replicate (*n* = 10 total per treatment) were randomly selected for carcass analysis. Following a 12-hour fasting period with ad libitum water access, birds were humanely euthanized via cervical dislocation. Carcass traits were evaluated using standardized protocols: dressing percentage was calculated as (eviscerated carcass weight / live body weight) × 100, with eviscerated weight determined after removal of feathers, head, feet, and all visceral organs (excluding kidneys). Giblet yield (heart + liver + gizzard) was expressed as a percentage of live weight: (giblet weight / live weight) × 100. All measurements were recorded using a calibrated digital balance (±0.1 g precision) and performed by trained personnel to ensure consistency (Islam et al., 2022).

### Gut histomorphology

Gut integrity and histomorphological parameters were systematically evaluated to assess dietary treatment effects. Immediately post-slaughter, standardized 2 cm segments of the mid-jejunum (collected at a fixed distance of ∼2 cm distal to the duodenal-jejunal junction for consistency) and cecum (proximal portion) were collected under aseptic conditions (Asghar et al., 2024; Kalsoom et al., 2024). Tissue samples were gently flushed with ice-cold phosphate-buffered saline (PBS, 0.1 M, pH 7.4) to remove luminal contents, then fixed in 10 % neutral buffered formalin (NBF) for 48 hours at 4°C to ensure optimal tissue preservation. Following fixation, specimens underwent sequential dehydration in an ethanol gradient (70 %, 80 %, 95 %, and 100 %), clearing in xylene, and paraffin embedding using standard histological protocols. Using a precision rotary microtome (Leica RM2235, or equivalent), 5 µm thick transverse sections were obtained and mounted on poly-L-lysine coated slides. Sections were stained with hematoxylin and eosin (H&E) following established protocols, with particular attention to staining consistency across all samples. Histomorphometric analysis was performed using a calibrated light microscope (Nikon Eclipse E200, or equivalent) at 400× magnification, with ten intact, well-oriented villi and associated crypts measured per sample. Villus height (VH) was measured from the crypt-villus junction to the villus tip, while crypt depth (CD) was measured from the base to the crypt-villus junction. The villus height-to-crypt depth ratio (VH:CD) was subsequently calculated. Digital images were captured using an integrated camera system (e.g., Nikon DS-Fi3) and analyzed using ImageJ software (v1.53) with appropriate calibration for scale (Islam et al., 2024).

### Statistical analysis

Data were analyzed using one-way analysis of variance (ANOVA) in SPSS software (Version 25). Differences between means were determined using Tukey’s post hoc test at a significance level of *P* < 0.05. Results were presented as means ± standard deviation (SD).

## Results

The effect of mealworm and superworm meal supplementation on the feed intake (FI) of Japanese quails is presented in Table 3. The results indicate significant differences in feed intake among the treatment groups from the second week onwards (*P* < 0.05). During the first week, no significant differences were observed in feed intake among the groups (*P* = 0.106). However, from the second week onwards, the control group exhibited the highest feed intake, while the MW+SW group recorded the lowest feed consumption. By the second week, the control group had the highest FI (69.00 ± 0.88 g), followed by the SW group (66.00 ± 0.57 g) and MW group (66.00 ± 1.15 g), while the MW+SW group had the lowest FI (60.66 ± 0.88 g) (*P* = 0.001). During the third week, a significant decline in FI was noted in the supplemented groups compared to the control group. The MW+SW group had the lowest FI (80.33 ± 0.88 g), whereas the highest intake was recorded in the control group (96.00 ± 0.57 g) (*P* < 0.05). In the fourth week, the control group continued to have the highest FI (138.00 ± 0.57 g), while the MW+SW group again had the lowest FI (127.67 ± 0.33 g) (*P* < 0.05). Similarly, in the fifth week, FI remained highest in the control group (161.00 ± 0.57 g) and lowest in the MW+SW group (154.00 ± 0.57 g) (*P* < 0.05). Overall, cumulative FI followed a similar trend, with the control group consuming the most feed (506.33 ± 2.33 g), followed by the SW group (491.00 ± 1.00 g), the MW group (481.67 ± 0.00 g), and the MW+SW group with the least intake (466.33 ± 1.85 g) (*P* < 0.05). These results suggest that insect meal supplementation influenced feed consumption, with the MW+SW combination diet resulting in the lowest feed intake compared to other groups.

**Table 3:** Effect of meal worm and super worm meals supplementation on feed intake (g) of Japanese quails.

Groups	1^st^ week	2^nd^ week	3^rd^ week	4^th^ week	5^th^ week	overall
Control	42.00 ± 1.15	69.00^a^ ± 0.88	96.00^a^ ± 0.57	138.00^a^ ± 0.57	161.00^a^ ± 0.57	506.33^a^ ± 2.33
MW	39.00 ± 0.57	66.00^b^ ± 1.15	86.66^c^ ± 0.88	131.00^c^ ± 0.57	159.00^b^ ± 0.00	481.67^c^ ± 0.00
SW	40.00 ± 2.08	66.00^b^ ± 0.57	92.66^b^ ± 0.88	135.00^b^ ± 0.57	157.33^b^ ± 0.88	491.00^b^ ± 1.00
MW+SW	43.00 ± 0.33	60.66^c^ ± 0.88	80.33^d^ ± 0.88	127.67^d^ ± 0.33	154.00^c^ ± 0.57	466.33^d^ ± 1.85
P value	0.106	0.001	0.000	0.000	0.000	0.000

Different superscripts in the same column with values ranging from a to d indicate a significant difference (*P* < 0.05).

MW = 3g/kg meal worm, SW = 3g/kg super worm; MW + SW = 3g/kg meal worm +3g/kg super worm.

The effect of mealworm and superworm supplementation on body weight gain (BWG) of Japanese quails is presented in Table 4. The results showed significant differences in BWG among the treatment groups from the second week onwards (*P* < 0.05). During the first week, no significant differences in BWG were observed among groups (*P* = 0.115). However, in the second week, quails in the MW+SW group exhibited the highest BWG (28.33 ± 1.52 g), which was significantly greater than the other groups (*P* = 0.012). In the third week, BWG remained highest in the MW+SW group (31.33 ± 0.33 g), while the lowest BWG was observed in the control group (26.83 ± 0.44 g) (*P* = 0.015). By the fourth week, quails fed the MW+SW diet continued to show the highest BWG (40.66 ± 0.88 g), while the control and MW groups had lower BWG values (*P* = 0.0125). During the fifth week, BWG was highest in the MW+SW group (50.66 ± 0.66 g), followed by the MW group (48.33 ± 0.33 g), while the control group had the lowest BWG (44.83 ± 1.09 g) (*P* = 0.006). Overall, cumulative BWG was significantly higher in the MW+SW group (165.00 ± 0.57 g), followed by the MW group (153.17 ± 0.44 g), SW group (151.00 ± 0.57 g), and the control group (147.00 ± 0.57 g) (*P* < 0.05). The feed conversion ratio (FCR) of Japanese quails supplemented with mealworm (MW), superworm (SW), and their combination (MW+SW) is presented in Table 5. The data reveal significant differences in FCR across treatment groups, particularly in later weeks of the experiment. In the first week, no significant differences (*P* > 0.05) were observed among the treatment groups. However, in the second week, the MW+SW group exhibited the lowest FCR (2.14 ± 0.03), significantly better (*P* < 0.05) than all other groups, while the control group had the highest FCR (3.13 ± 0.09). Similarly, during the third week, the MW+SW group showed the most efficient feed conversion (2.56 ± 0.05), followed by MW (3.05 ± 0.04) and SW (3.41 ± 0.20), with the control group having the highest FCR (3.58 ± 0.75).

**Table 4:** Effect of supplementation of meal worm and super worm meals on body weight gain (g) of Japanese quails.

Groups	1^st^ week	2^nd^ week	3^rd^week	4^th^ week	5^th^ week	overall
Control	17.00 ± 0.57	22.16^b^ ± 0.44	26.83^b^ ± 0.44	36.16^b^ ± 0.92	44.83^c^ ± 1.09	147.00^d^ ± 0.57
MW	15.00 ± 0.57	25.00^b^ ± 0.57	28.33^b^ ± 0.33	36.50^b^ ± 0.28	48.33^ab^ ± 0.33	153.17^b^ ± 0.44
SW	13.33 ± 0.88	24.33^b^ ± 1.76	27.33^b^ ± 1.45	38.66^ab^ ± 0.88	47.00^bc^ ± 1.00	151.00^c^ ± 0.57
MW+SW	14.00 ± 0.22	28.33^a^ ± 1.52	31.33^a^ ± 0.33	40.66^a^ ± 0.88	50.66^a^ ± 0.66	165.00^d^ ± 0.57
P value	0.115	0.012	0.015	0.0125	0.006	0.00

Different superscripts in the same column with values ranging from a to d indicate a significant difference (*P* < 0.05).

MW = 3g/kg meal worm, SW = 3g/kg super worm; MW + SW = 3g/kg meal worm +3g/kg super worm.

**Table 5:** Effect of supplementation of meal worm and super worm meals on Feed conversion ratio of Japanese quails.

Groups	1^st^ week	2^nd^ week	3^rd^ week	4^th^ week	5^th^ week	overall
Control	2.47 ± 0.07	3.13^a^ ± 0.09	3.58^a^ ± 0.75	3.82^a^ ± 0.09	3.59^a^ ± 0.09	3.44^a^ ± 0.02
MW	2.60 ± 0.12	2.64^b^ ± 0.01	3.05^ab^ ± 0.04	3.58^ab^ ± 0.02	3.29^b^ ± 0.02	3.14^b^ ± 0.03
SW	3.03 ± 0.32	2.73^b^ ± 0.18	3.41^b^ ± 0.20	3.49^b^ ± 0.08	3.35^b^ ± 0.08	3.25^c^ ± 0.01
MW+SW	3.19 ± 0.35	2.14^c^ ± 0.03	2.56^c^ ± 0.05	3.14^c^ ± 0.07	3.04^c^ ± 0.04	2.82^d^ ± 0.02
P value	0.215	0.001	0.001	0.001	0.03	0.00

Different superscripts in the same column with values ranging from a to d indicate a significant difference (*P* < 0.05).

MW = 3g/kg meal worm, SW = 3g/kg super worm; MW + SW = 3g/kg meal worm +3g/kg super worm.

The trend continued in the fourth and fifth weeks, where the MW+SW group consistently demonstrated the lowest FCR values (3.14 ± 0.07 and 3.04 ± 0.04, respectively), indicating superior feed efficiency compared to the other groups. The control group, on the other hand, maintained the highest FCR throughout the experimental period. The overall FCR results confirm that quails supplemented with MW+SW (2.82 ± 0.02) had significantly improved feed efficiency compared to MW (3.14 ± 0.03), SW (3.25 ± 0.01), and the control group (3.44 ± 0.02). No mortality was reported in the experiment.

Table 6 presents the effect of mealworm (MW) and superworm (SW) supplementation on the dressing percentage and giblet percentage of Japanese quails. The dressing percentage varied significantly (*P* = 0.000) among the different dietary treatments. The control group exhibited the lowest dressing percentage (60.54 ± 0.34 %), while the highest dressing percentage was observed in quails fed a combination of MW and SW (66.66 ± 0.46 %). Quails supplemented with MW alone (63.11 ± 0.51 %) showed a significantly higher dressing percentage than those in the control group, whereas SW supplementation alone (62.03 ± 0.49 %) resulted in an intermediate dressing percentage. These results suggest that dietary supplementation with MW and SW, either alone or in combination, enhances carcass yield in Japanese quails, with the combination group (MW+SW) showing the most pronounced improvement.

**Table 6:** Effect of supplementation of meal worm and super worm meals on dressing percentage of Japanese quails.

Groups	Dressing percentage	Giblet percentage
Control	60.54^c^ ± 0.34	6.78 ± 0.04
MW	63.11^b^ ± 0.51	6.80 ± 0.03
SW	62.03^bc^ ± 0.49	6.81 ± 0.05
MW+SW	66.66^a^ ± 0.46	6.84 ± 0.06
P-value	0.000	0.12

Different superscripts in the same column with values ranging from a to d indicate a significant difference (*P* < 0.05).

MW = 3g/kg meal worm, SW = 3g/kg super worm; MW + SW = 3g/kg meal worm +3g/kg super worm.

No significant differences (*P* = 0.12) were observed in giblet percentage across treatment groups. The giblet percentage remained relatively stable among the different dietary treatments, ranging from 6.78 ± 0.04 % in the control group to 6.84 ± 0.06 % in the MW+SW group. These findings indicate that while insect meal supplementation influences dressing percentage, it does not significantly impact gib Table 7 presents the effects of mealworm (MW) and superworm (SW) supplementation on the histomorphological parameters of Japanese quails. The parameters assessed include villus height (VH), villus width (VW), crypt depth (CD), and the villus height-to-crypt depth ratio (VH:CD), which are critical indicators of gut health and functionality.

**Table 7:** Effect of supplementation of meal worm and super worm meals on histomorphology of Japanese quails.

Group	Villus height (µm)	Villus width (µm)	Crypt depth (µm)	VH:CD
Control	342.47^d^ ± 1.40	101.50^d^ ± 0.78	137.20^a^ ± 0.96	2.49^d^ ± 0.01
MW	366.86^b^ ± 0.91	106.48^b^ ± 0.70	113.76^c^ ± 0.25	3.22^b^ ± 0.03
SW	362.93^c^ ± 0.96	104.51^c^ ± 0.42	117.52^b^ ± 0.61	3.08^c^ ± 0.01
MW+SW	378.22^a^ ± 1.41	109.88^a^ ± 0.20	110.37^d^ ± 0.57	3.42^a^ ± 0.01
P-value	0.000	0.000	0.000	0.000

Different superscripts in the same column with values ranging from a to d indicate a significant difference (*P* < 0.05).

MW = 3g/kg meal worm, SW = 3g/kg super worm; MW + SW = 3g/kg meal worm +3g/kg super worm.

The supplementation of MW, SW, and the combination of both (MW+SW) significantly increased the villus height compared to the control group (*P* = 0.000). The control group showed the lowest villus height (342.47 ± 1.40 mm), while the highest value was observed in quails fed with the combination of MW and SW (378.22 ± 1.41 mm). The MW group (366.86 ± 0.91 mm) and SW group (362.93 ± 0.96 mm) showed significantly higher villus heights compared to the control group, but their values were lower than the MW+SW group. These results suggest that insect supplementation can improve villus growth, with the combination of MW and SW providing the most significant enhancement in villus height.

Villus width followed a similar trend to villus height, with a significant increase observed in the groups supplemented with MW, SW, and MW+SW (*P* = 0.000). The control group had the smallest villus width (101.50 ± 0.78 mm). The highest villus width was observed in the MW+SW group (109.88 ± 0.20 mm), followed by the MW group (106.48 ± 0.70 mm) and the SW group (104.51 ± 0.42 mm). The increase in villus width suggests that insect-based diets contribute positively to the structural integrity of the gut mucosa in Japanese quails, which may aid in improving nutrient absorption.

The crypt depth varied significantly among the groups (*P* = 0.000). The control group exhibited the deepest crypts (137.20 ± 0.96 mm), indicating a possible compensatory mechanism for the poor gut health associated with the standard diet. However, the groups supplemented with MW, SW, and MW+SW all exhibited significantly lower crypt depths. The MW group had a crypt depth of 113.76 ± 0.25 mm, the SW group showed 117.52 ± 0.61 mm, and the MW+SW group had the lowest crypt depth (110.37 ± 0.57 mm). A reduction in crypt depth is typically associated with enhanced villus growth and better gut health, suggesting that insect meal supplementation improved the gut architecture of quails. The VH:CD ratio is an important indicator of intestinal health, reflecting the balance between villus growth and crypt depth. Significant differences were observed across the treatments (*P* = 0.000). The control group had the lowest VH:CD ratio (2.49 ± 0.01), which reflects the compromised gut architecture under normal feeding conditions. The groups receiving insect supplementation showed a substantial improvement in this ratio, with the MW+SW group achieving the highest VH:CD ratio (3.42 ± 0.01). The MW group (3.22 ± 0.03) and the SW group (3.08 ± 0.01) also showed improved ratios compared to the control, but their values were lower than the combination group. These findings highlight that insect-based diets, particularly the combination of MW and SW, contribute significantly to improving the gut morphology, which could have implications for enhancing nutrient absorption and overall quail performance.

## Discussion

Poultry production is an efficient and cost-effective method for providing animal protein, offering rapid production of meat and eggs with excellent feed-to-protein conversion efficiency (Abudabos et al., 2013, 2017a,b; Mirnawati et al., 2023; Dinasarki et al., 2024). Traditionally, the poultry industry has relied heavily on plant-based protein sources, particularly soybean meal, to meet the protein requirements of poultry diets (Uzair et al., 2025; Islam et al., 2024). However, the rising cost of these conventional feed ingredients and the potential for future shortages could pose significant challenges to the sustainability of poultry production (Dabbou et al., 2021). This situation has prompted researchers to explore alternative protein sources that are not only cost-effective but also sustainable. Insects, particularly mealworms and superworms, have emerged as promising alternatives due to their rich nutritional profiles, including essential amino acids, fatty acids, and minerals, making them a viable option for livestock and poultry feeding (Benzertiha et al., 2020).

The inclusion of mealworm and superworm meals in the diets of Japanese quails significantly influenced both total and weekly feed consumption. Notably, the control group exhibited the highest cumulative feed intake, whereas the group supplemented with both mealworm and superworm meals demonstrated the lowest. This reduction may relate to the higher protein and lipid content of insect meals, which can promote satiety. In addition, chitin present in insect exoskeletons, although often beneficial for gut health, may slightly reduce digestibility, contributing to lower intake. Interestingly, these findings contrast with some studies in broilers (Islam and Yang, 2017; Sedgh-Gooya et al., 2022), suggesting species-specific responses to insect supplementation.

Body weight gain was significantly enhanced in quails receiving insect meals, with the highest gain observed in the combined mealworm + superworm group. This improvement is likely linked to the high-quality protein and balanced amino acid profiles of insect meals, which support muscle development and efficient nutrient utilization. Our results align with Benzertiha et al. (2020) and Yildirim et al. (2020), though differences with Dabbou et al. (2021) highlight the influence of diet formulation and insect processing methods.

Feed conversion ratio (FCR) was also markedly improved by insect supplementation, particularly at the highest inclusion levels. Enhanced FCR indicates more efficient nutrient utilization, making insect meals an attractive option for improving production economics. Similar findings have been reported in broilers and quails (Islam and Yang, 2017; Zadeh et al., 2019; Bovera et al., 2016). Differences across studies may reflect variation in insect meal processing (e.g., defatting, chitinase pretreatment), which strongly influences digestibility (Gasco et al., 2019).

Dressing percentage was significantly improved in insect-fed groups, consistent with previous findings (Bovera et al., 2016; Khan et al., 2022). The favorable fatty acid profile of mealworms and superworms may improve carcass composition and meat yield, further enhancing their value as feed ingredients. Giblet yields remained unaffected, supporting the safety and neutrality of insect meals on internal organ development.

Gut histomorphology was positively influenced by insect supplementation, as evidenced by greater villus height, width, and VH:CD ratio. These improvements suggest enhanced nutrient absorption and intestinal health, consistent with earlier studies (Zadeh et al., 2019; Khan et al., 2022). Contrasting findings in broilers (Dabbou et al., 2021) suggest that effects may depend on species, inclusion level, or processing of insect meals.

Beyond biological effects, the economic feasibility of insect meals warrants consideration. Although insects are nutritionally comparable to soybean meal, large-scale production costs, processing requirements (e.g., drying, defatting), and regulatory frameworks currently limit their affordability. However, with technological advancements and waste-to-feed approaches (e.g., using agro-industrial by-products as substrates), production costs are expected to decline, making insect meals more competitive. For commercial adoption, evaluating the cost-benefit ratio relative to conventional protein sources is essential.

## Conclusion

The inclusion of mealworm and superworm meals in quail diets significantly influenced key production parameters, including feed intake, body weight gain, and feed conversion efficiency. Notably, the combination diet comprising both insect meals (MW+SW) resulted in reduced feed intake while achieving superior weight gain, improved feed efficiency, enhanced carcass yield, and favorable gut histomorphology. These outcomes indicate that insect-based diets, particularly when combining mealworm and superworm meals, can serve as a promising alternative protein source in quail nutrition, supporting both performance and health-related parameters.

## Disclosure statement

No potential conflict of interest was reported by author(s).

## Ethical approval statement

The Committee on Animal Rights and Welfare, The University of Agriculture, Peshawar, Pakistan approved this study (FAHVS/122/2023).

## AI information

Language was corrected with the help of AI (chatgpt)

## CRediT authorship contribution statement

**Hanan Al-Khalaifah:** Resources. **Zeeshan Ahmad:** Data curation. **Rafi Ullah:** Supervision, Software. **Ziaul Islam:** Visualization, Validation, Investigation. **Asad Sultan:** Formal analysis. **Ziaul Islam:** Investigation. **Ala Abudabos:** Software. **Shabana Naz:** Writing – review & editing, Writing – original draft. **Rifat Ullah Khan:** Writing – review & editing, Writing – original draft. **Ibrahim A. Alhidary:** Writing – review & editing, Writing – original draft.

## Disclosures

Authors declare no conflict of interest.

## Data Availability

Data will be made available from the authors upon reasonable request.

## References

[bib0002] Abudabos A.M., Samara E., Hussein E.O., Al-Atiyat R.M., Al-Haidary A. (2013). Influence of stocking density on welfare indices of broilers. Ital. J. Anim. Sci..

[bib0003] Abudabos A.M., Al-Atiyat R.M., Stanley D., Aljassim R., Albatshan H.A. (2017). The effect of corn distiller’s dried grains with solubles (DDGS) fortified with enzyme on growth performance of broilers. Environ. Sci. Pollut. Res..

[bib0004] Abudabos A.M., Aljumaah R.S., Algawaan A.S., Al-Sornokh H., Al-Atiyat R.M. (2017). Effects of hen age and egg weight class on the hatchability of free range indigenous chicken eggs. Rev. Bras. Cienc. Avic..

[bib0001] Ajmal A.S., Hussain Z., Jalees M.M., Shafi J., Manzoor S., Haq A.U. (2023). Performance of broiler birds on feeding natural anti-stressors in summer during heat stress. Asian J. Agric. Biol..

[bib0005] Al-Suwailem N.K., Kamel N.N., Abbas A.O., Nassar F.S., Mohamed H.S., Gouda G.F., Safaa H.M. (2024). The impact of dietary Moringa oleifera leaf supplementation on stress markers, immune responses, and productivity in heat-stressed broilers. Int. J. Vet. Sci..

[bib0006] Asghar T., Mohiuddin M., Mohiuddin A., Mansoor M.K., Siddique A., Habib M., Kamal T., Hussain R., Ghori M.T., Rizwana H., Abid I., Shabbir A. (2024). Hemato-biochemical changes, molecular characterization and phylogenetic analysis of the 2022 lumpy skin disease outbreak in Cholistan. Pakistan. Asian J. Agric. Biol..

[bib0007] Benzertiha A., Kierończyk B., Kołodziejski P., Pruszyńska-Oszmałek E., Rawski M., Józefiak D., Józefiak A. (2020). Tenebrio molitor and Zophobas morio full-fat meals as functional feed additives affect broiler chickens’ growth performance and immune system traits. Poult. Sci..

[bib0008] Biasato I., Gasco L., De Marco M., Renna M., Rotolo L., Dabbou S., Schiavone A. (2018). Yellow mealworm larvae (Tenebrio molitor) inclusion in diets for male broiler chickens: effects on growth performance, gut morphology, and histological findings. Poult. Sci..

[bib0009] Boonmee T., Jaikan W., Brokner C., Blanch A., Khajarern J. (2024). Efficacy of enzyme-treated soybean meal on broiler performance, nutrient digestibility, and carcass quality. Int. J. Vet. Sci..

[bib0010] Bovera F., Loponte R., Marono S., Piccolo G., Parisi G., Iaconisi V., Gasco L., Nizza A. (2016). Use of Tenebrio molitor larvae meal as protein source in broiler diet: effect on growth performance, nutrient digestibility, and carcass and meat traits. J. Anim. Sci..

[bib0011] Dabbou S., Lauwaerts A., Ferrocino I., Biasato I., Sirri F., Zampiga M., Schiavone A. (2021). Modified black soldier fly larva fat in broiler diet: effects on performance, carcass traits, blood parameters, histomorphological features and gut microbiota. Animals.

[bib0012] Devi P.C., Mirnawati, Marlida Y. (2023). The combination of Bacillus subtilis with Lactobacillus fermentum in improving the quality and nutrient contents of fermented palm kernel meal. Int. J. Vet. Sci..

[bib0013] Dinasarki D., Tenrisanna V., Amrawaty A.A. (2024). Broiler product quality: the global scientific research landscape and implications for marketing performance. Int. J. Agric. Biosci..

[bib0014] Dragojlović D., Đuragić O., Pezo L., Popović L., Rakita S., Tomičić Z., Spasevski N. (2022). Comparison of nutritional profiles of super worm (Zophobas morio) and yellow mealworm (Tenebrio molitor) as alternative feeds used in animal husbandry: is super worm superior?. Animals.

[bib0016] Elahi U., Xu C.C., Wang J., Lin J., Wu S.G., Zhang H.J., Qi G.H. (2022). Insect meal as a feed ingredient for poultry. Anim. Biosci..

[bib0017] Flis M., Czyżowski P., Rytlewski G., Grela E.R. (2024). Insect meal as a dietary protein source for pheasant quails: performance, carcass traits, amino acid profile and mineral contents in muscles. Animals.

[bib0018] Gasco L., Biasato I., Dabbou S., Schiavone A., Gai F. (2019). Animals fed insect-based diets: state-of-the-art on digestibility, performance and product quality. Animals.

[bib0019] Habib M.A., Salma U., Amin M.N., Rahman M.G., Haque M.A. (2024). The effect of halquinol on growth performance, carcass traits, and blood-lipid profile in broiler chickens. Int. J. Agric. Biosci..

[bib0020] Hafeez A., Akram W., Al-Khalaifah H., Naz S., Khan R.U., Tufarelli V., Alhidary I.A. (2025). Enzyme inclusion or fermentation of canola-based diets generate different responses in growth indicators, carcass quality, nutrient digestibility, bone strength, and blood biochemical parameters in broiler chickens. Arch. Anim. Breed..

[bib0021] Islam M.M., Yang C.J. (2017). Efficacy of mealworm and super mealworm larvae probiotics as an alternative to antibiotics challenged orally with Salmonella and E. coli infection in broiler chicks. Poult. Sci..

[bib0022] Islam Z., Sultan A., Khan S.Z., Khan S.B. (2022). Effect of organic acids blend, microencapsulated phyto-essential oils individually or in combination on growth performance, gut health and nutrients utilization of broilers. Pak. J. Zool..

[bib0023] Islam Z., Sultan A., Khan S., Khan K., Jan A.U., Aziz T., Alasmari A.F. (2024). Effects of an organic acids blend and coated essential oils on broiler growth performance, blood biochemical profile, gut health, and nutrient digestibility. Ital. J. Anim. Sci..

[bib0024] Kalsoom R., Asfour H.Z., Ali H.M., Qayyum A., Anjum S., Maqbool F., Sial N., Hussain R., Alamri S.H., Ali N., Rajeh N., Irshad I., Idrees A. (2024). Bifenthrin induced toxic effects on haematological, reproductive and histo-morphological profile in adult male quail (Coturnix japonica). Asian J. Agric. Biol..

[bib0025] Khan K., Al-Khalaifah H., Ahmad N., Khan M.T., Alonaizan R., Khan R.U., Naz S., Abudabos A., Alhidary I.A. (2025). Dietary supplementation of cinnamon and turmeric powder enhances growth performance, nutrient digestibility, immune response, and renal function in broiler chickens. Poult. Sci..

[bib0026] Khan S., Tanweer A.J., Rafiullah I., Abbas G., Khan J., Imran M.S., Kamboh A.A. (2022). Effect of supplementation of mealworm scales (Tenebrio molitor) on growth performance, carcass traits and histomorphology of Japanese quails. J. Anim. Health Prod..

[bib0027] Magnoli A.P., Parada J., Luna M.J., Corti M., Escobar F.M., Fernández C., Coniglio M.V., Ortiz M.E., Wittouck P., Watson S., Cristofolini L.A., Cavaglieri L. (2024). Impact of probiotic saccharomyces cerevisiae var. Boulardii RC009 alone and in combination with a phytase in broiler chickens fed with antibiotic-free diets. Agrobiol. Rec..

[bib0029] Mirnawati, Ciptaan G., Martaguri I., Ferawati, Srifani A. (2023). Improving quality and nutrient content of palm kernel meal with Lactobacillus fermentum. Int. J. Vet. Sci..

[bib0030] Nouri H.S.A., Ali S.A.M., Abdalla H.O., Ahmed H.B. (2024). Performance and carcass characteristics of broiler chickens kept on heated soybean meal. Agrobiol. Rec..

[bib0031] Othman M.F., Chung A.Y.K., Halim R.M., Kalil M.S., Bakar N.A., Aziz A.A. (2024). Valorization and optimization of protein in fermented palm kernel cake: influence on broiler chicks growth. Int. J. Agric. Biosci..

[bib0032] Pietras M., Orczewska-Dudek S., Szczurek W., Pieszka M. (2021). Effect of dietary lupine seeds (Lupinus luteus L.) and different insect larvae meals as protein sources in broiler chicken diet on growth performance, carcass, and meat quality. Livest. Sci..

[bib0033] Rasool A., Qaisrani S.N., Khalique A., Hussain J. (2023). Insoluble fiber source influences performance, nutrient digestibility, gut development and carcass traits of broilers. Pak. J. Agric. Sci..

[bib0034] Sarsembayeva N., Abdigaliyeva T., Omarkulova Z., Kauymbayeva M., Ustenova G., Lozowicka B., Yefremov S., Ibragimov P. (2025). Effect of primary and active shungite on the quality of feed, meat, and eggs of broilers. Int. J. Vet. Sci..

[bib0035] Sedgh-Gooya S., Torki M., Darbemamieh M., Khamisabadi H., Abdolmohamadi A. (2022). Growth performance and intestinal morphometric features of broiler chickens fed on dietary inclusion of yellow mealworm (Tenebrio molitor) larvae powder. Vet. Med. Sci..

[bib0037] Shaukat N., Farooq U., Akram K., Shafi A., Hayat Z., Naz A., Hakim A., Hayat K., Naseem S., Khan M.Z. (2023). Antimicrobial potential of banana peel: a natural preservative to improve food safety. Asian J. Agric. Biol..

[bib0038] Sultan A., Aziz T., Islam Z., Uzair M.S., Alhindary I.A., Khan R.U., Tiwari R. (2024). Effect of ginger-based zingibain enzyme on growth and intestinal health in Japanese quails. Arch. Anim. Breed..

[bib0039] Sultana M.A., Habib M.A., Amin M.N., Sabuz S.H., Salma U., Begum M.D., Haque M.A. (2025). Effects of yogurt on growth performance, carcass traits, lipid profile and fecal microbial load of broiler chickens. Int. J. Agric. Biosci..

[bib0040] Uzair M.S., Sultan A., Islam Z., Shah M., Tahir M., Naz S., Khan R.U. (2025). Efficacy of zingibain phyto-protease on growth performance, litter quality and gut microbiota in broilers fed high animal protein concentrates. Ital. J. Anim. Sci..

[bib0041] van Huis A. (2021). Prospects of insects as food and feed. Org. Agric..

[bib0042] Yildirim U., Sarica S., Kanoglu B. (2020). Defatted yellow mealworm larvae (Tenebrio molitor L.) meal as possible alternative to fish meal in quail diets. S. Afr. J. Anim. Sci..

[bib0043] Zadeh Z.S., Kheiri F., Faghani M. (2019). Use of yellow mealworm (Tenebrio molitor) as a protein source on growth performance, carcass traits, meat quality and intestinal morphology of Japanese quails (Coturnix japonica). Vet. Anim. Sci..

